# Agency in Fertility Decisions in Western Europe During the Demographic Transition: A Comparative Perspective

**DOI:** 10.1007/s13524-016-0536-0

**Published:** 2017-01-09

**Authors:** David Sven Reher, Glenn Sandström, Alberto Sanz-Gimeno, Frans W. A. van Poppel

**Affiliations:** 1 Grupo de Estudios Población y Sociedad (GEPS), Universidad Complutense de Madrid, Madrid, Spain; 2 Department of Historical, Philosophical and Religious Studies and the Centre for Demographic and Ageing Research (CEDAR), Umeå University, Umeå, Sweden; 3 Netherlands Interdisciplinary Demographic Institute (NIDI)/Royal Netherlands Academy of Arts and Sciences (KNAW), The Hague, The Netherlands

**Keywords:** Demographic transition, Fertility, Mortality, Sex-preferences, Europe

## Abstract

We use a set of linked reproductive histories taken from Sweden, the Netherlands, and Spain for the period 1871–1960 to address key issues regarding how reproductive change was linked specifically to mortality and survivorship and more generally to individual agency. Using event-history analysis, this study investigates how the propensity to have additional children was influenced by the number of surviving offspring when reproductive decisions were made. The results suggest that couples were continuously regulating their fertility to achieve reproductive goals. Families experiencing child fatalities show significant increases in the hazard of additional births. In addition, the sex composition of the surviving sibset also appears to have influenced reproductive decisions in a significant but changing way. The findings offer strong proof of active decision-making during the demographic transition and provide an important contribution to the literature on the role of mortality for reproductive change.

## Introduction

This study addresses the role of childhood mortality for reproductive decision-making during the demographic transition. It is widely held that before the transition, reproductive decisions tended to be made at a societal or group level, often by means of changes in marriage timing and intensity (Mason 1997). However, as the transition progressed, reproductive decisions became increasingly individual- and family-based, responding to concrete conditions of families more than to accepted societal norms (Reher 2011). From this perspective, the demographic transition can be viewed as a key episode in the progress of human agency, central to all processes of modernization. Showing the existence of active decision-making during the transition empirically, especially with respect to the importance of mortality change for reproductive decisions, however, has not been a simple matter: until fairly recently, this type of decision-making was more basic postulate than a proven cornerstone of transition theory. In this article, we marshal important new empirical evidence, enabling us to view this issue from a different perspective and leading to a nuanced view of the importance of agency and how it changed over the transition.

The role of mortality has long been central to the conception of the demographic transition, mostly because of the major changes in mortality during this period. Inherent in Notestein’s (1945) original formulation of demographic transition theory, especially as subsequently modified by Kingsley Davis (1963) and Ansley Coale (1973, 1986), is the role of mortality change as a key factor triggering fertility decline in the late nineteenth century, both at a societal and a familial level (Reher 2004). In Coale’s formulation of this issue, the decline of childhood mortality was a necessary precondition for any sustained decline in marital fertility because it led to the perception that reduced fertility was advantageous (see Mason 1997:446–447; see also Kirk 1996), although at a societal level, other, more general factors might cloud this intrinsic link (Coale 1973:54, 62–66; Coale 1986). Showing that survival outcomes were a key part of reproductive decision-making is a way of underscoring the importance of choice and human agency during the transition. Underlying this argument is the supposition that couples generally desired a given number of surviving children, which tended to be small, as evidenced by prevailing growth rates prior to the demographic transition. This cornerstone of transition theory subsequently came under severe criticism, even as other scholars have insisted on its validity.^1^

**Fig. 1:**
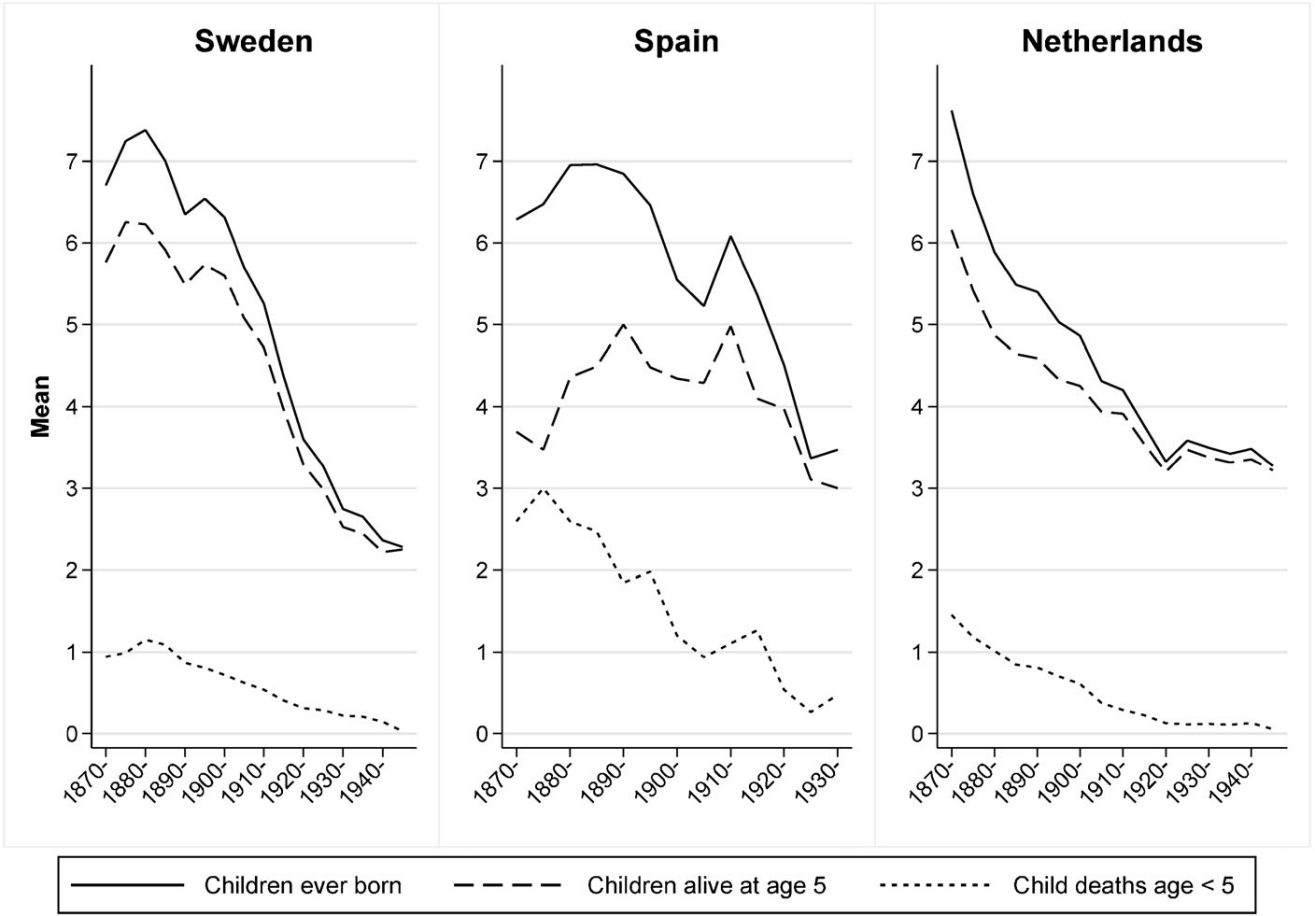
Mean number of children ever born and indicators of child survival in Sweden, Spain, and the Netherlands by marriage cohorts 1870–1949. Mean is based on couples observed until the end of their reproductive history. Cohorts married in 1930–1949 are aggregated for Spain. *Source:* Sweden: POPLINK Database, Demographic Database, Umeå University (2015). Spain: Aranjuez Civil Registers. Netherlands: Historical Sample of the Netherlands (HSN)

A number of studies making use of longitudinal data have addressed this issue (Alter 1988; Bengtsson and Dribe 2006; Knodel 1988; Van Bavel 2003, 2004; Van Bavel and Kok 2010). This study follows this line of research and builds directly on the results of three recent publications that have addressed this issue specifically by using longitudinal microdata (Reher and Sanz-Gimeno 2007; Reher and Sandström 2015; Van Poppel et al. 2012). In these articles, linked reproductive histories over the period of the demographic transition for a Spanish sample (town of Aranjuez) and a Dutch sample were used to study the extent to which mortality and mortality change were factors in fertility limitation. Here, we extend this approach by modeling how both childhood mortality and the sex composition of the surviving sibset influenced fertility decisions in three different contexts in Western Europe during the historical fertility decline. The countries chosen for analysis are Sweden, the Netherlands, and Spain. Although these countries showed marked variation in cultural and economic structures, all experienced a rapid decline in both childhood mortality and fertility starting in the late nineteenth century that continued at an increased pace during the first decades of the twentieth century. During the first part of the period under study, the Swedish and the Dutch settings appear to be at a more advanced stage of the demographic transition; by the end, however, prevailing levels of fertility and mortality are quite similar. Figure 1 shows the reproductive experience in each setting for couples married 1870–1949 measured as the mean number of fatalities of children under age 5, the mean number of children surviving to age 5, and the mean number of children ever born. Despite some differences, the similarity in the general trends is evident during the period covered by the figure.

When addressing the connection between childhood mortality and fertility outcomes, it is important to differentiate between several possible mechanisms by which declining mortality can influence fertility. Previous literature has often highlighted three types of factors, two of which are pertinent for the issues at hand (see, e.g., Palloni and Rafalimanana 1999). First increased child survival has an individual-level biological effect in terms of longer periods of breast-feeding when infants survive. If lactation is not interrupted because of infant death, postpartum infecundity is prolonged and thus tends to delay conception even in the absence of active contraception (Knodel and Van de Walle 1967). This effect would exist independent of any desire for fertility limitation, and therefore choice is not involved.

Second, individual-level behavioral effects operate when couples have preferences regarding family size and at least partially effective means of adjusting fertility. These preferences are a response to the number of surviving children rather than to the number of children ever born, using two types of strategy. One is a short-term strategy designed to replace a child who has just died. This sort of child replacement can be found in many populations past and present, although in the past, it is very difficult to distinguish it from the mechanical biological response to the early cessation of breast-feeding described earlier. Beyond this immediate effect, if couples had fertility goals and the ability to implement them, their fertility decisions would tend to be based on the overall survival status of their sibset rather than solely on the outcome of the previous birth. Both child replacement and reactions to the overall number of surviving children are indicators of the existence of fertility goals—although in the first case, it would be at least partly in response to short-term goals together with the biological effects mentioned earlier.

Another related mechanism is an insurance or a hoarding effect (Preston 1978; see also Alter 1988). When mortality is high and variable, prudent couples will try to overshoot their actual target family size to ensure a minimum number of surviving children that eventually reach adulthood. When levels of childhood mortality decrease sufficiently, the need for this type of insurance behavior decreases, and couples can choose to both stop and space their births at lower parities given that they are confident that most, or all, of their children will survive to adulthood.

Although the biological or lactation effect should be found mainly in a natural fertility setting, both the replacement effect and the hoarding effect should be present when fertility control, albeit inefficient, is a realistic possibility as long as childhood mortality is high and unstable enough to make hoarding a sensible strategy. We can expect that the ability of people to influence their reproductive outcomes efficiently improved as the demographic transition progressed and as stopping behavior became widely adopted (Anderton and Bean 1985; Gillis and Tilly 1992:15). Whenever possible, researchers should distinguish between these different forms of short-term and long-term behavioral responses to childhood mortality.

When prevailing fertility is relatively high, the link between childhood mortality and fertility (especially with respect to stopping behavior) can be bidirectional. Although the number of surviving offspring may influence fertility choices, it is also true that the level and the timing of fertility can in itself be a cause of childhood mortality (Knodel 1988; Van de Kaa 1996). For example, the children of highly fecund women will tend to experience relatively higher levels of mortality that can be attributed to shorter birth intervals, to a reduction in the time parents (mothers) spend with each child, and to greater levels of maternal depletion (Oris et al. 2004). Researchers should consider this sort of reverse causality and control for differences in fecundity between different couples.

This analysis also addresses the impact of the sex composition of the surviving sibset at the time of different reproductive decisions. This subject is less central to the literature on the demographic transition but can be interpreted as another indicator of choice and human agency during this period. Boys and girls filled different economic, social, and cultural roles within the household, all of which may have influenced fertility decisions. Some recent studies found evidence that a lack of male offspring leads to an increased propensity for additional childbearing compared with couples with mixed-sex sibsets or only girls in Germany (Sandström and Vikström 2015), the United States (Bohnert et al. 2012), and Spain (Reher and Sandström 2015) during the fertility transition. To the best of our knowledge, this study is the first comparative analysis regarding the role of sex preferences in culturally different contexts in Europe for the period covering the demographic transition.

## Methodological Considerations

### Data: Origins, Characteristics, and Quality

Our study is based on three data sources, all of which include micro data of individual reproductive histories, even though their origins are not the same. We discuss the specifics of the different sources in the following paragraphs. All three data sets are based on linked vital registration sources: for example, parish records, civil registers with which individuals can be followed over time in terms of births, in- and outmigration, marriages, deaths, and their links to other family members. For the Dutch data, the individuals are followed in the sources also after out-migration across areas of the Netherlands, which explains the much longer follow-up time in the Dutch case as opposed to Sweden, and in particular, the Spanish data. For Sweden and Spain, the couples are censored at their first out-migration from the studied areas. All data sources contain socioeconomic information of at least the occupation held by the individual at the time of vital events, and in some cases at more frequent intervals based on censuses or catechetical examination registers. For matters of comparability, we have chosen to follow couples married during 1870–1949 from their first marriage until they are censored because of outmigration, divorce, death of one of the spouses, the woman reaching menopause at age 50, or our observation window ends in 1960. Table 1 gives information about the total number of cases and basic information about observation time and number of births while under observation.^2^

**Table 1:** Size and characteristics of Swedish, Spanish, and Dutch samples

	Sweden	Spain	The Netherlands
Total Number of Couples Observed	24,065	3,484	3,394
Number of Births While Under Observation	86,450	13,443	14,435
Total Time at Risk in Years	383,931	39,817	82,526
Mean Time at Risk in Years	15.9	11.42	24.31

*Sources:* Sweden: POPLINK Database, Demographic Database, Umeå University (2015). Spain: Aranjuez Civil Registers. The Netherlands: Historical Sample of the Netherlands (HSN).

#### Dutch Data

The Dutch sample used here (described in depth in Van Poppel et al. (2012)) is based on two databases that enable us to follow reproductive patterns over a prolonged period. First, the sample includes full maternity histories of couples born between 1850 and 1922 who gave birth between 1870 and 1970, based on consecutive series of municipal population registers and its successors. These data make it possible to reconstruct complete life histories of couples from 1850 to the end of the registers in 1939. Second, after 1939, the bound population registers were replaced by personal record cards, containing largely the same information as the population register. Data from the registers, personal record cards, and personal record lists were collected within the framework of the Historical Sample of the Netherlands (HSN), a database with information on the complete life histories of a national random sample of the 1850–1922 birth cohorts in the Netherlands (Mandemakers 2002).

#### Spanish Data

The Spanish data are described in detail in Reher and Sanz-Gimeno (2007) and Reher and Sandström (2015). We take advantage of data collected from civil registration records for the Spanish town of Aranjuez, located near Madrid. Our study covers the fertility development of couples living in the town between 1870 up until 1960, when the youngest women included in the sample reached menopause and a time when the population of the town more than tripled from around 8,000 inhabitants and the weight of agriculture in the urban economy diminished substantially. The database used for our analysis comprises individual biographies that have been constructed using family reconstitution methods using Civil Registration records as well as six household listings conducted in the town (1877, 1903, 1915, 1945, 1960, and 1975).

#### Swedish Data

The Swedish data is based on the POPLINK database covering approximately 350,000 individuals born from the late eighteenth century until the 1970s living in the northern coastal region of Sweden in Västerbotten County (Westberg et al. 2016). The POPLINK database contains individual life biographies, family relations (parents-children, spouses) and all vital events of the individuals living in the included parishes. The source also holds extensive longitudinal socioeconomic information regarding occupational status updated from the information found in the catechetical examination registers. The parishes are distributed throughout the coastal region of Västerbotten and include more than two-thirds of the population in the province during the nineteenth and twentieth centuries. Economic development presents the typical distinctive traits of most Swedish regions—that is, being mainly rural until the early twentieth century and thereafter characterized by industrialization and a growing public sector. Västerbotten conforms closely to the national trends in fertility and mortality but with somewhat higher levels of completed fertility compared with the Swedish national average.

### Statistical Method

For the analysis, we use event-history methods in terms of nonparametric survival estimates and Cox proportional hazards regressions to estimate the influence of childhood mortality and the sex composition of the surviving children in a univariate as well as multivariate context. This approach enables us to account simultaneously for differences in both the pace and the propensity of having an additional child, given the survivorship and sex composition of the children at earlier parities at any given time during the reproductive history. Using event-history methods that handle right-censoring during the period at risk maximizes the number of observations and enables us to follow couples married up until relatively recent periods (1949).^3^

To test the statistical significance of child fatalities in a univariate setting, we use Kaplan-Meier estimates of the probability of an additional birth and log rank tests of the equality of the survivor function for parities 2–6. In the multivariate analysis, we model the effect of child survival and the sex composition of the surviving children by using both single-failure (parities 2–5) and multiple-failure (parities 3–8) Cox proportional hazards regressions. The propensity to have additional children is modeled dependent on the number of surviving children and the sex composition of the surviving children in the household at time *t*. The analysis starts when couples become at risk of confinement of the second child in the case of the single-failure specification and at child 3 in the multiple-failure analysis.

All multivariable models include a time-invariant control for the socioeconomic position (SES) of the father at the time of marriage (or first child birth in a minority of the cases from Aranjuez) in order to adjust our estimates for possible SES differences in birth intensities. Coding of the occupational titles was done according to the HISCO classification system (Van Leeuwen and Maas 2011), which was then stratified according to the Social Power coding scheme developed by Van de Putte and Miles (2005). A control for the 10-year marriage cohort of the couple is included in all models to adjust for increasing fertility control over time. Two groups of marriage cohorts (pre- and post-1900), corresponding roughly to the early stages of the fertility transition and to a period in which fertility was falling substantially in all countries, are also used to study change over time.

To adjust for underlying fecundity differences among couples, all models include a control for the quartiles of birth interval for child 1→2. Our premise is that women having a faster pace of childbearing will also have higher childhood mortality because of maternal depletion and/or resource competition. Here, the birth interval between the first and second child has been used as a proxy for biological differences in fecundity because any fertility control at this early stage of reproduction should have been low. All references to parity in this article refer to the crude parity equal to the number of children ever born. Thus, we compare only those women who have had the same number of children exposed to the risk of dying at any given point of their reproductive lives.

To adjust for the possibility of premature termination of breast-feeding, we use a time-varying indicator variable set to 1 in the interval 9–12 months after a child younger than 12 months dies. The variable is then reset to 0 if childbirth occurs during the interval or if the 12 months limit is reached before another birth occurs. This approach limits the effect of the variable to the period when it is most likely that a possible termination of breast-feeding could influence the fecundity of the woman. This is the same method as used in a number of other fertility studies applying event-history analysis (see, e.g., Alter 1988; Amialchuk and Dimitrova 2012). This strategy does not discriminate between a biological effect of truncated breast-feeding and a replacement response to infant mortality in the very early stages of life. However, controlling for this timespan will decrease the possibility of biological factors influencing the estimate of our main childhood mortality variable. We tested different lengths for the breast-feeding indicator, and overall results remained the same even if we assume that all conceptions occurring up to nine months after the death of an infant are generated by a biological rather than a behavioral effect of child mortality. In the case of the pooled analysis that includes all cases from the three countries in a joint data set, we include a country variable as an additional stratifying variable to adjust for differences in the baseline hazards across countries. Further, in the pooled analysis, we weight Swedish, Dutch, or Spanish cases to achieve estimates in which each country has the same impact on parameters despite the differences in sample size.

The outcome measure in hazard regression is sensitive to both timing and incidence and will thus reflect differences in both spacing and stopping behavior. We make no attempt to differentiate between the two distinct forms of intentional fertility control in this study. Our aim here is to test whether birth intensities were influenced by the number of surviving children and the sex composition of the surviving sibset across culturally and economically different settings in Europe during the fertility transition. We interpret this as an indication that the couple implemented reproductive strategies to achieve a target family size and/or a particular sex composition among the surviving children.

In both single- and multiple-failure models, we treat the number of surviving children and the sex composition as time-varying covariates that are recalculated if the death of a sister or brother occurs during the time at risk. In episodes where all children are deceased, this is treated as having a mixed sex composition. This approach enables us to estimate the effect of child deaths and sex composition on birth intensities for families with different experiences of child survival and sex composition among the surviving children.

In the single-event models, the effect of declining fecundity as a function of the age of the mother is not estimated with a control variable in the models. Rather, we choose to let this effect be part of the baseline hazard by using the attained age of the mother as analysis time. This approach has been shown to more accurately control for the effect of the age of an individual when becoming at risk than using time-on-study as analysis time and controlling for age at baseline with a covariate (Cheung et al. 2003; Korn et al. 1997; Thiébaut and Bénichou 2004). Consequently, in the hazard regression analysis, the women do not enter the analysis at time 0 but enter the risk set for parity 2, 3, . . . 8 at their attained age with late entry/left truncation in both the single- and multiple-failure models.

For the single-event models, we analyze the transitions at parity 2–5 separately for each parity and apply a standard proportional hazards model as suggested by Cox (1972), allowing for a time-varying definition of childhood mortality and sex composition. Consequently, the notation for the proportional hazards regression function with time-varying covariates is as follows:

$$ h\left(t,x(t),\upbeta \right)={h}_0(t) \exp \left[x\hbox{'}(t)\upbeta \right], $$

where the function *h*_0_(*t*) denotes the baseline hazard function that characterizes how the hazard changes as a function of analysis time *t*, which in our specification equals the attained age of the mother. The function exp(*x*' (*t*)β) characterizes how the hazard changes as a function of the subject covariate values at time *t* since becoming at risk. Each parity transition is estimated as a separate process with separate models for each parity.

In the multiple-failure specification, we model the effect of the independent variables over the full reproductive history from becoming at risk of having a third child until either parity 8 is reached or the couple is censored for other reasons. We start the analysis when the couple becomes at risk for having a third child because at least two children are needed in order to differentiate between couples that have a mixed sex composition of offspring and those with only boys or only girls. By implication, this also means that only fertile couples are used in this analysis.

The specification of the multiple-failure process is implemented according to the conditional counting process approach developed by Prentice et al. (1981). The notation for the multiple-failure hazard regression function is as follows:

$$ h\left(t,x(t),s,\upbeta \right)={h}_{0s}(t) \exp \left[x\hbox{'}(t)\upbeta \right]. $$

This model is conditional in the sense that subjects are not assumed to be at risk of having a subsequent event until a prior event has occurred. The stratum variable *s* is used to keep track of the event/risk set that the subject belongs to at each point of analysis time, and each event consequently has a separate event-specific baseline hazard. Onset of risk occurs when the woman has her second child and becomes at risk of giving birth to a third child. Subjects enter the risk sets for the following events 4–8 at the time they experience the previous event through late entry, and the clock is not reset. Follow-up time is thus broken into segments defined by the events, where each event is treated as a separate risk set defined by the stratum variable *s*, which allows the baseline hazard to be separate for each event (birth) across the reproductive history. This approach allows us to achieve a model of the hazard of additional births over the full course of the recurrent event process (Hosmer et al. 2008:294–296). Intrasubject clustering of the hazard across events experienced by the same woman is accounted for by applying a sandwich (robust) estimator to the standard errors of the parameters. Model specifications with an additional time scale for time since last birth (gap time model) as well as model with a gamma frailty on the level of the family to account for unmeasured heterogeneity yielded no differences in hypothesis tests and were therefore discarded for parsimony.

For a discussion of different approaches to recurrent event models see, for example, Hosmer et al. (2008:287–296), Kalbfleisch and Prentice (2002:279–299), and Prentice et al. (1981). We calculated all estimates with the collection of *st* commands for event-history analysis available in Stata 13.1 (StataCorp 2013).

## Results

**Fig. 2:**
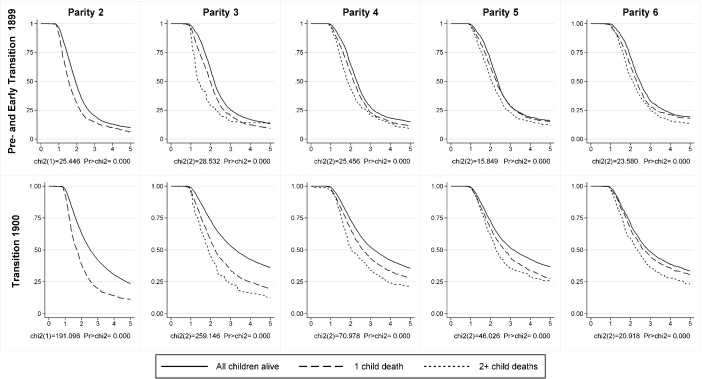
Proportion not progressing to next birth by cumulative number of child fatalities and time at risk in years since last birth. Kaplan-Meier survival functions and log-rank tests for parity 2–6, marriage cohorts 1870–1949, in pooled sample for Sweden, Spain, and the Netherlands. Cases are weighted to adjust for the different sample sizes in the separate countries. *Sources:* Sweden: POPLINK Database, Demographic Database, Umeå University (2015). Spain: Aranjuez Civil Registers. Netherlands: Historical Sample of the Netherlands (HSN)

Figure 2 shows the probability of not having had a child of parity *n* for the pooled sample including Sweden, Spain, and the Netherlands as described by nonparametric Kaplan-Meier survival estimates for parities 2–6 for couples married before the turn of the century (1870–1899) and after (1900–1949). Separate survival curves for couples having different numbers of surviving children at time *t* since becoming at risk of having a child of parity *n* are given, as are log-rank tests of difference in survival between the groups*.* It is clear that couples who experience child fatalities have substantially higher risks of progressing to the next parity for all parities until child 6 and that the effect is especially strong for the lower parities. The differences in the survival are significant below the 1 % level for all parities. Wilcoxon and Tarone-Ware tests that give greater weight to events occurring early when more subjects remain at risk as opposed to late during the period at risk yield the same results regarding significance (Hosmer et al. 2008:44–59).

Based on a comparison between the two panels showing the association before and after 1900, it is clear that the strength of the association increased as the fertility transition progressed. In the period after 1900, the tendency for increased stopping at the lower parities (2–4) is limited almost entirely to the couples not experiencing child fatalities. Couples with child fatalities during the transitional phase after 1900 tend to have more or less the same progression probabilities as similar couples in the pretransitional period. Combining the data from all three countries, we find that in the pretransitional period, more than 50 % of the couples experienced the death of at least one child under age 5. By the 1930s and 1940s, this proportion had dropped to well below 10 % in Sweden and in the Netherlands and was only slightly higher in Spain.^4^ Consequently, decreased childhood mortality shifted larger and larger proportions of couples out of a category exhibiting more or less pretransitional progression ratios into a category exhibiting a new low-fertility behavior. These results suggest that fertility would not have decreased nearly as much had mortality remained at the high levels observed during the nineteenth century.

In the multivariate analysis shown in Table 2, we test the extent to which our preliminary conclusion changes when we control specifically for the early termination of lactation as well as a number of other possible confounders. Here, we estimate single-failure Cox regression models for parities 2–5 for the pooled sample in order to ascertain the effects of child survival and sex composition of the surviving sibset when we control for the socioeconomic position of the father, marriage cohort, the age of the mother when becoming at risk at different parities, and the birth interval between first and second child (a proxy for underlying fecundity). We also estimate a control variable treating the 9–12 months after the death of an infant separately in order to adjust our main childhood mortality variable for the possibility of a biological or lactation effect on fertility. Once again, we divide the analysis into two periods to assess the effect of the independent variables during the earlier and more advanced stages of the fertility transition. Diagnostics of the models show no signs of nonproportionality, misspecification, or unduly influential cases indicating that model estimates are robust.

**Table 2:** Cox proportional hazard regressions. Relative hazard of reaching parity 2–5 for marriage cohorts 1870–1900 and 1901–1949 in Sweden, Spain, and the Netherlands^a^

Variables	Categories	Parity 2		Parity 3		Parity 4		Parity 5	
		–1899	1900–	–1899	1900–	–1899	1900–	–1899	1900–
Socioeconomic Position of the Father at the Time of Marriage (SOCPO)	Unskilled workers	1	1	1	1	1	1	1	1
	Semiskilled workers	1.08	0.83**	1.03	0.96	1.06	0.91	1.03	0.93
	Skilled workers	0.96	0.83**	1.06	0.89**	0.97	0.89*	0.99	0.95
	Middle class: Farmers	1.00	1.11**	1.12**	1.12**	1.02	1.20**	1.04	1.14**
	Middle class	0.90^†^	0.75**	1.00	0.84**	0.88^†^	0.86**	0.92	0.88^†^
	Elite	0.70**	0.85**	0.87	0.88*	0.88	0.86^†^	1.00	1.08
	No information	0.93	1.07*	0.99	1.03	0.95	0.97	0.98	1.01
Total Number of Child Deaths at Time *t*	All children alive	1	1	1	1	1	1	1	1
	1 deceased child	1.28**	1.48**	1.10*	1.24**	1.09*	1.14**	1.05	1.10*
	2 deceased children			1.21	1.59**	1.17*	1.29**	1.19**	1.42**
	3 or more deceased					1.28	1.30	1.43**	0.89
Lactation Indicator Month 9–12 After Infant Death	No	1	1	1	1	1	1	1	1
	Yes	2.27**	2.58**	2.39**	2.63**	2.94**	3.68**	2.70**	3.35**
Sex Composition of Surviving Children at Time *t*	Mixed	––	––	1	1	1	1	1	1
	No surviving girls	––	––	1.06	1.06*	0.97	1.03	0.90*	0.99
	No surviving boys	––	––	1.11*	1.06^†^	1.01	1.03	1.00	1.14*
Number of Observations		7,173	24,899	6,803	19,252	6,228	12,927	5,586	8,501
Chi-Square		147.799	627.296	496.427	2,042.387	319.974	745.650	210.627	281.483
Prob. > Chi-Square		.000	.000	.000	.000	.000	.000	.000	.000

*Notes:* Coefficients are shown in exp(β) form. Models include controls for 10-year marriage cohort, quartiles of birth interval for child 1–2, and country.

^a^Models are weighted to adjust the different sample sizes in the countries.

^†^ *p* < .10; *p < .05; **p < .01

The substantial effect on further childbearing of the number of surviving children is clear in the multivariate models, even when we control for other important influences on fertility that might confound the effect of childhood mortality. The relative importance of individual biological versus behavioral effects of childhood mortality is specified by including a control variable for the deaths of infants, although it is important to note that this variable will include both the mechanical effect of terminated breast-feeding as well as any deliberate replacement behavior that the death of the previous child might cause during the period covered by the control variable. Completely disentangling these two effects with the available data is not possible.

Even with these adjustments included, we find strong and significant effects of childhood mortality on birth intensities, with approximately a 40 % to 60 % increase in the hazard of additional births among the couples who experienced the most unfavorable child survival outcomes. As expected, the effect of childhood mortality is substantially larger in the period after 1900, when fertility control had become more widespread, with the relative hazard for the childhood mortality variable approximately doubled in size compared with the earlier period. When running models in which the period is interacted with the child mortality variable, we find that the interaction is positive and significant (*p* <.001) in the separate countries as well as for the pooled sample. This result suggests that the effects of child mortality increased during the latter part of the transition. We also find a shift in the effect over the reproductive span in terms of being larger at the lower parities (especially progression 2→3). The effect increases more or less linearly as a function of the number of child fatalities. In sum, couples experiencing childhood deaths had a significantly higher number of children ever born compared with those with a more favorable mortality experience, even when we account for a number of possible confounders.

Our models also show that couples reacted to having children of only one sex by adjusting their fertility in order to have additional offspring of the desired sex. Significant changes in the way couples responded to the sex composition of their surviving sibsets appear to have taken place over time. Before 1900, it was more important for couples to have surviving male offspring, while the lack of surviving girls had little impact on fertility decisions at the lower parities. The results mirror the preference for male children also found in Germany during the early stages of the fertility transition (Sandström and Vikström 2015) and in the United States by Bohnert et al. (2012). As the countries entered a more advanced stage of the transition after 1900, this preference for boys among couples with family sizes of two to three children appears to have changed, and the lack of girls also resulted in higher birth intensities. In other words, we see a shift from a boy preference to a more symmetrical preference during the twentieth century, which is a pattern of behavior that has also been found for Germany (Sandström and Vikström 2015). Couples with large families during the fertility decline continued to express a boy preference at higher parities. The upcoming country-specific analysis will reveal interesting country differences in the effect of sex composition that shed additional light on the issue of changes over time in the relative value of male and female children.

We now turn to the issue of country-specific patterns in the effect of childhood mortality and sex composition over time. To investigate these issues, we pool the events over parities 3–8 and estimate multiple-failure Cox regression models to achieve higher statistical power and the ability to estimate more extensive models. The results are shown in Table 3, which contains eight models. Models 1–2 give the average effect on birth intensities for parities 3–8 for all couples in the pooled sample before and after 1900. Models 3–8 give country-specific estimates for the pre- and post-1900 periods used here.

**Table 3:** Cox proportional hazard regressions for progression to parities 3–8 (multiple failure per subject model): Marriage cohorts 1870–1949 in Sweden, Spain, and The Netherlands

Variables	Categories	Pooled^a^		Sweden		Spain		Netherlands	
		–1899	1900–	–1899	1900–	–1899	1900–	–1899	1900–
Socioeconomic Position of the Father at the Time of Marriage (SOCPO)	Unskilled workers	1	1	1	1	1	1	1	1
	Semiskilled workers	1.02	0.93^†^	1.03	0.94*	1.14	0.98	0.98	0.94
	Skilled workers	0.98	0.93*	0.99	0.85**	0.99	0.89	0.94	0.96
	Middle class: Farmers	1.03	1.22**	1.07**	1.23**	1.10	0.90	0.97	1.18*
	Middle class	0.88**	0.92*	0.87**	0.87**	0.92	1.04	0.84**	0.93
	Elite	0.74**	0.94	0.70**	0.90*	1.15	0.86	0.70*	0.98
	No information	0.98	1.03	1.06	0.98	1.02	1.02	0.70	1.04
Total Number of Child Deaths at Time *t*	All children alive								
	1 deceased child	1.05*	1.12**	1.04*	1.11**	0.99	1.14**	1.10*	1.12*
	2 deceased children	1.09**	1.21**	1.07*	1.11**	1.06	1.38**	1.11	1.26*
	3 or more deceased children	1.17**	1.05	1.09^†^	0.98	1.13	1.29*	1.12	0.89
Lactation Indicator Month 9–12 After Infant Death	No								
	Yes	3.03**	3.51**	3.10**	3.62**	3.13**	3.32**	2.87**	4.06**
Sex Composition of Surviving Children at Time *t*	Mixed								
	No surviving girls	1.01	1.06**	0.98	1.04*	1.03	0.98	1.04	1.14**
	No surviving boys	1.06*	1.08**	1.05*	1.08**	1.12*	1.10*	1.01	1.08
Number of Observations		31,111	52,959	22,203	39,995	4,678	6,387	4,230	6,577
Chi-Square		937.227	1,022.072	2,021.639	2,359.117	293.194	244.588	213.359	204.515
Prob. > Chi-Square		.000	.000	.000	.000	.000	.000	.000	.000

*Notes:* Models include controls for 10-year marriage cohort and quartiles of birth interval for child 1–2. The pooled model includes the country as an additional stratifying variable.

^a^Pooled models are weighted to adjust the different sample sizes in the countries.

^†^ *p* < .10; *p < .05; **p < .01

These models confirm and reinforce the parity-specific results found in the earlier models. In all three settings, the effects of childhood survival for reproductive choice tend to increase during the latter period when fertility control becomes more widespread. The increase is most substantial in Spain, which is the country with the largest relative decrease in childhood mortality (see Fig. 1). These results suggest that differences in childhood mortality had a substantial impact on the decision to continue having additional children across very different settings in Europe during the fertility decline. We find a clear and statistically significant behavioral component of childhood mortality during the course of the fertility transition that remains even when we control for other possible confounders. Comparing models with and without a lactation control (results available from the authors upon request) reveals that the introduction of the control variable reduces the effect of the main mortality variable by less than 50 %. Given that the indicator for the infant death of the preceding child also at least partly reflects behavioral adjustments of fertility caused by the death of the previous child, we can conclude that the biological mechanism is substantially less important for reproductive outcomes than the behavioral responses to childhood mortality.

The increased effect of childhood mortality in the latter period after 1900, when survivorship increased and fertility declined rapidly, can be explained by two factors at work during the demographic transition. First, the ability of couples to express preferences in response to differences in child survival improved during the transition because they were increasingly ready, willing, and able to control their fertility, thus fulfilling the classical norms mentioned years ago by Ansley Coale as preconditions for the onset of conscious fertility control (Coale 1973). Second, as childhood mortality decreased rapidly during the same period that fertility decline accelerated, losing one or even two children increasingly became an exceptional—and therefore inadmissible—experience for couples. Consequently, the death of a child became a much more important event for the parents who experienced it than it was during the nineteenth century, when most couples must have expected to lose one or more of their children (Reher and González-Quiñones 2003). It is not surprising that the behavioral response to the loss of a child increased in magnitude during the latter period—a time when the death of a child had become a much more uncommon event, and the ability to regulate fertility had improved.

Looking at our other indicator of agency in fertility decisions, prior to 1900, couples having no surviving boys were, on average, significantly more prone to have an additional child than those having children of both sexes or no surviving girls. Because contemporary medical research has conclusively shown no association between fecundity and the probability of having a child of either sex (Eisenberg et al. 2011; Joffe et al. 2007), we can assume that any differences in birth intensities between couples lacking either girls or boys were the result of preferences regarding the sex composition of the surviving offspring.

Different from the similar effects of childhood mortality across the settings included here, we find substantial variations in the effect of sex composition when we analyze the countries separately. Sweden shows the same pattern as that found in Germany (Sandström and Vikström 2015) during the transition. Here, having no surviving boys significantly increases birth intensities among couples married before 1900, whereas this pattern shifts to a symmetrical preference for boys and girls among couples married after 1900. This shift indicates an increase in the relative value put on girls by parents that coincided with the shift toward smaller families during the advanced stages of the fertility transition in Sweden as well as Germany.

Spain and the Netherlands also exhibit variations in fertility patterns according to the sex composition of the surviving children but rather differently from Sweden. For Spain, the results from Reher and Sandström (2015) are confirmed in that we find no shift to a symmetrical preference; however, we do find clear indications of a decreased strength in the male preference. Additional analysis by parity (results available from authors upon request) reveal that the male preference is least visible among couples marrying after 1930 and among those with smaller families, thus suggesting similar changes afoot in Spain toward the end of the period.^5^

The Dutch sample also shows a different pattern of behavior. Surprisingly, we see no indications of boys being preferred over girls, even at very early stages of the transition during the nineteenth century. As in Sweden, there was a substantial increase in the impact of sex composition between the period before and after 1900; but in the Netherlands, it is primarily couples lacking surviving girls that exhibit increased birth intensities. The tendency after 1900, on the other hand, is clearly toward a more symmetrical pattern given that the effect for having no surviving boys is also positive although not statistically significant. The difference in significance for this parameter in Sweden and the Netherlands is related to the smaller sample size in the Dutch case. In sum, the stronger tendency for preferring girls in the surviving sibset in the Netherlands is a very interesting finding that merits further enquiry in terms of how to explain this early onset of a girl preference in the Dutch case.

The most important finding, however, is that the results across all three countries for both sex composition and childhood mortality can be considered examples of how agency influenced fertility outcomes even at the early stages of the fertility transition. The results also illustrate how these influences increased in importance as couples acquired the means to regulate their fertility more successfully over time as parity-dependent control became widespread. Also of interest is that the sex composition of children had a significant impact on birth intensities in both Sweden and Spain in the period before 1900, when a substantial fertility decline had not yet occurred. This result affords additional proof that even during the earlier period, couples were reacting to certain outcomes by regulating their fertility decisions at a time when average family size still remained at its pretransitional levels.

## Concluding Discussion

This study examines the role of choice and human agency during the demographic transition. In so doing, it joins a growing body of literature aimed at reevaluating the role of childhood mortality for reproductive change. The results shown here are striking and promise to stimulate debate in the field.

The role of mortality in the demographic transition has a rather checkered history. Originally a cornerstone of demographic transition theory, its importance ended up being considered almost anecdotal due mainly to a plethora of studies stemming directly or indirectly from the European Fertility Project.^6^ It is our contention that this misperception was due to the use of inadequate data because the only way to show how mortality affected reproduction was by means of linked micro data. In this study, we used massive databases taken from three European countries to show how the number of surviving children was an important predictor of the likelihood of having additional children. We used sophisticated statistical techniques to show a pattern of behavior that was also visible with straightforward univariate analysis. When controlling for a whole host of social, demographic, and biological factors, we found significance levels for this effect that remained high. These links were present in the early transitional period but increased in strength as the transition progressed. In other words, as the transition progressed, the loss of a child became increasingly rare but also increasingly important in determining reproductive choice. The results were visible in the pooled multivariate models and present in every setting, despite relatively different positions of each setting with respect to the transition itself. In sum, these results offer convincing proof that reduced childhood mortality was linked to lower fertility during the fertility transition in Europe.

Whether mortality change was the key factor or only a key factor triggering the transition itself is not entirely clear from the results presented here. A straightforward decomposition exercise appears to suggest that the massive reduction in childhood mortality taking place in the period under study in itself tended to push fertility downward as more and more couples had positive experiences with the survival of their children. Yet, couples were increasingly efficient in limiting their fertility beyond, or perhaps in addition to, the cascading decreases in mortality. Eventually, however, defending a reasonable net family size was replaced by the goal of smaller families. During the period under study, the Dutch and Swedish couples were well along this path, with Spanish couples lagging behind but moving in the same direction.

In this article, the nexus between mortality and fertility was shown to be very close and laced with cultural and economic preferences for gender composition within the sibset. Here, our different data sets show different results. Before 1900, couples in Sweden and Spain showed a clear male preference: couples without boys were more likely to bear additional children. This did not appear to be the case in the Netherlands, however. It is tempting to relate this to the idea that from the seventeenth and eighteenth centuries onward, the Dutch Republic provided more economic and social space for women than in other European societies, leading to a relatively more emancipated position (De Moor and Van Zanden 2015; De Vries and Van der Woude 1997; Schama 1987). After 1900, there was a clear shift to valuating female children more favorably in both the Netherlands and Sweden. In Spain, however, a male preference remains evident, illustrating that the gender regime in this southern part of Europe did not change as rapidly and early as in other regions of the continent. Even in Aranjuez, Spain, however, there is some indication that male preference tended to decline in the cohorts married after 1930 and among those with relatively small families, thus suggesting that an increase in the relative value put on girls by parents may be an interesting byproduct of modernization processes everywhere.^7^ These disparities in the timing and intensity of changes in gender preferences may well be related to the long-standing existence of strong and weak family systems in different parts of Europe partly characterized by the relative visibility of women in society (Reher 1998). More research on the pervasive influence of the family during this period is needed before the importance of issues like these can be ascertained.

Given the individual level link between child mortality and subsequent fertility behavior uncovered in our results, it is difficult not to agree with Mason’s (1997: 446) conclusion that any viable transition theory must acknowledge that “without a mortality decline, a fertility decline is highly unlikely.” Although in principle, a direct causal relationship between mortality and fertility can by proven only by means of a randomized experiment, an advantage of the longitudinal methods and data used here is that individual couples can be followed over time. This approach enables us to examine their fertility behavior after they have been able to observe how many of their earlier offspring had survived and the specific sex composition of these surviving offspring. The empirical results of these models have shown that indeed these variables were important for the fertility of couples later in their reproductive lives. The fact that we included measures of exposure and outcome across the life course adds strength to a causal interpretation of the results. In doing this, we attempted to control as much as possible for factors that might affect both child mortality and fertility, such as the age of the woman, fecundity, and breast-feeding. Previous research has shown that sex-selective infanticide was not a pertinent feature of the pretransitional European fertility regime (for a review, see Lynch 2011). This makes our sex composition variable especially useful for identifying active decision-making because we can assume that exposure is assigned by random biological processes. Therefore, exposure to different sex compositions comes close to having the characteristics of a natural experiment. We found that the sex of children did influence future reproductive decisions but that it was much less important than childhood survival for parental decision-making.

Ultimately, this study shows that choices were indeed being made during the demographic transition in Sweden, the Netherlands, and Spain. Because these choices were predicated on the size and sex composition of surviving sibsets, it is difficult to escape the fact that they are examples of active decision-making. During the early transitional period, this practice was halting and perhaps less efficient than it might have been. Later, however, this was all to change as the evidence presented here shows. In the Netherlands and in Sweden, net family size was falling for marriage cohorts from the start of the century, while this was the case by the 1930s in Spain. After this happens, ideational changes related to, for example, increasing investments in the human capital of children and increasing opportunity costs for women as they are integrated into market work outside the family will result in further decreases in the demand for children (Reher 2011).

Consequently, this entire process did not take place in a vacuum, and it is not our intention to assert that, say, having a child was determined solely by the number of surviving children. Other forces of change at work were in the process of reshaping society and how people perceived themselves, their lives, and their ability to make important choices—reproductive choices, in this case. Education, social, and cultural change, technological innovation, and attitudes toward life are major forces in this process of modernization that may well have influenced both childhood survival and reproductive choice at the same time. These are the nuts and bolts of the historical processes taking place during this period. Underneath this general process, however, people were making decisions based on the survival of their offspring, but these decisions were made amid other, more general causes. In many ways, the changes in fertility behavior uncovered in this article speak to the larger forces of societal change taking place alongside the specific dimensions of choice analyzed here. Both patterns are complementary and both deserve the active attention of researchers.

## Footnotes

1 For a detailed appraisal of this debate, see Reher and Sandström (2015) and Van Poppel et al. (2012).

2 We considered using the German village genealogy data set generated by John Knodel but opted not to do so because the Knodel data cover only the early stages of the fertility transition until approximately 1900.

3 Sample selections differ somewhat from the one used in Reher and Sandström (2015) that focused on the Spanish data. Slight differences in the estimates for Spain compared with Reher and Sandström (2015) are explained by the fact that cohorts born later during the twentieth century are now also included.

4 These differences are the result of a higher prevalence of larger families in the earlier period and, more importantly, the massive changes over time in the probabilities of childhood death.

5 Both categories have a larger share of the sample in this analysis as opposed to the full reproductive histories chosen for analysis in Reher and Sandström (2015).

6 For recent contributions to this line of thought, see Perry (2014), Guinnane (2011), and Cummins (2012).

7 For more on sex preference and the tendency for symmetrical preferences in contemporary European countries and in the United States, see, for example, Pollard and Morgan (2002) and Mills and Begall (2010).
